# Efficiency of Interstellar Nanodust Heating: Accurate Bottom-up Calculations of Nanosilicate Specific Heat Capacities

**DOI:** 10.1021/acs.jpca.2c02199

**Published:** 2022-06-08

**Authors:** Joan Mariñoso Guiu, Stefan T. Bromley

**Affiliations:** † Departament de Ciència de Materials i Química Física & Institut de Química Teòrica i Computatcional (IQTCUB), Universitat de Barcelona, c/Martí i Franquès 1-11, 08028 Barcelona, Spain; ‡ Institució Catalana de Recerca i Estudis Avançats (ICREA), Passeig Lluis Companys 23, 08010 Barcelona, Spain

## Abstract

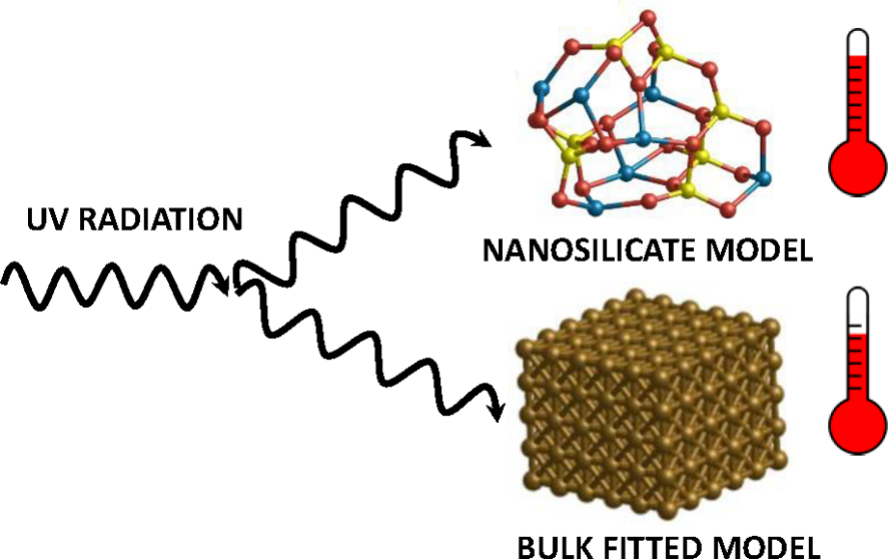

Ultrasmall nanosized silicate grains are likely to be highly abundant in the interstellar medium. From sporadically absorbing energy from ultraviolet photons, these nanosilicates are subjected to significant instantaneous temperature fluctuations. These stochastically heated nanograins subsequently emit in the infrared. Previous estimates of the extent of the heating and emission have relied on empirical fits to bulk silicate heat capacities. The heat capacity of a system depends on the range of available vibrational modes, which for nanosized solids is dramatically affected by the constraints of finite size. Although experimental vibrational spectra of nanosilicates is not yet available, we directly take these finite size effects into account by using accurate vibrational spectra of low-energy nanosilicate structures from quantum chemical density functional theory calculations. Our results indicate that the heat capacities of ultrasmall nanosilicates are smaller than previously estimated, which would lead to a higher temperature and more intense infrared emission during stochastic heating. Specifically, we find that stochastically heated grains ultrasmall nanosilicates could be up to 35–80 K hotter than previously predicted. Our results could help to improve the understanding of infrared emission from ultrasmall nanosilicates in the ISM, which could be observed by the James Webb Space Telescope.

## Introduction

Dust particles are prevalent throughout the interstellar medium (ISM), where they reveal themselves through their interaction with electromagnetic radiation.^1^ Much of the dust mass in the ISM is carried by grains with a typical radius (*r*) of ∼0.1 μm (100 nm). However, the number of dust grains with respect to size approximately follows an inverse power law (*n*_g_(*r*) = *cr*^–3.5^),^2^ which massively favors higher populations of smaller grains. It has long been realized that the low time-averaged temperature of very small grains in the ISM would not capture the large temperature fluctuations due to transient heating of such species when interacting with the radiation field of the ISM.^3^ The first calculations of this effect showed that dust grains with diameters of only a few nanometres are particularly susceptible to sporadic temperature spikes.^4^ This effect, now commonly referred to as stochastic heating, involves the relatively infrequent absorption of energy from ultraviolet (UV) photons, which leads to above average grain temperatures and a subsequently rapid readjustment to ambient ISM temperatures via emission in the infrared (IR). Stochastic heating is now appreciated to be an important mechanism for generating IR emission in a number of astrophysical environments. The temperature and emissivity of stochastically heated nanograins on the rates of UV energy absorption and subsequent IR emission, which, in turn, is intimately linked to their specific heat capacity.^5,6^ Most modern calculations of stochastic heating follow the approach by Draine and Li, which used a statistical mechanical treatment.^5^ In this work the temperature dependent heat capacity, *C*(*T*), of a nanosized dust grain is directly calculated from its distribution of available vibrational modes (for inducing temperature increase) with respect to their frequency (i.e., energy). We follow this approach and show how it can be enhanced for the smallest grain sizes by using vibrational modes evaluated from direct and accurate quantum chemical calculations using atomistically detailed stable nanograin structures.

Herein, we consider silicate dust, which is observed in a wide range of astronomical environments and typically has a magnesium-rich pyroxene (MgSiO_3_) or olivine (Mg_2_SiO_4_) composition.^7^ Considering observations of IR emission in the ISM, it has been estimated that ultrasmall nanosilicates (*r* ≤ 1.5 nm) could be highly abundant and account for ∼10% of all silicon in the dust population.^8^ Nanosilicates can be formed from the processing of larger grains in the ISM,^9^ where they are a likely source of the anomalous microwave emission.^10−12^ and can assist in the formation of H_2_^13,14^ and ices.^15^ With respect to the latter, studies have shown that stochastically heated silicate grains with *r* ≈ 0.005 μm (5 nm) exhibit temperature fluctuations up to 20–40 K, which is predicted to have a significant impact on their role in astrochemical processes.^16,17^ For even smaller nanosilicates possessing only a few 10s of atoms (approximately with *r* ≤ 0.5 nm), the fluctuations in temperature from stochastic heating have been estimated to be up to ∼1000 K^5^ with, as yet, unknown consequences for astrochemistry.

In ref (5) the heat capacities of nanosilicate grains were estimated based on a bimodal distribution of harmonic vibrations, which was found to be reasonable for reproducing measured temperature-dependent heat capacities of bulk basalt, while providing a somewhat poorer fit to bulk SiO_2_. This bulk approximation (hereafter referred to as the “bulk fitted model”, BFM) has since been employed in other studies of stochastic heating of small silicate dust grains.^16,17^ For nanosilicates, their extreme small size and resultant non-bulk-like and noncrystalline structures leads to their vibrational spectra being quite unlike those of bulk silicates.^18^ Thus, although the general BFM approach represents a solid microscopic framework for calculating heat capacities of small silicate dust grains, the original implementation has a few shortcomings: (1) The reference bulk materials (i.e., basalt, obsidian, silica) are not compositionally good matches for the composition of Mg-rich silicate dust grains in the ISM; (2) The fit of the spectrum to the bulk *C*(*T*) versus *T* curves is purely empirical and does not consider the direct physical origin of the vibrational modes in silicate dust grains; (3) The fit is with respect to the measured *C*(*T*) of bulk samples (i.e., not nanosized grains). Herein, we address all these three issues using an accurate quantum chemical approach in which we directly calculate the vibrational modes in atomically detailed and energetically stable nanosilicates having Mg-rich olivinic and pyroxenic compositions. As heat capacities of nanosilicates are not yet experimentally available, our approach provides a direct and accurate alternative to relying on empirical fits to bulk heat capacity data. Our resulting calculated heat capacities and energy-dependent temperatures of ultrasmall nanosilicate grains are compared with those derived from the BFM approximation.

In previous work, we derived the atomistically detailed structures of low energy nanosilicate clusters for astronomically relevant Mg-rich pyroxene and olivine compositions.^18^ Therein, we confirmed that the IR vibrational spectra of these species, accurately obtained using quantum chemical density functional theory (DFT) based calculations, are quite distinct from those of bulk silicates. Herein, we use DFT-based calculations to obtain accurate full vibrational distributions of selected nanosilicate grains and use this data to directly derive temperature-dependent heat capacities. Our results show that BFM estimates of the distribution of vibrational modes tend to overestimate the heat capacity of nanosilicate grains, especially at temperatures around 50–300 K. As a result, for a typical range of energies for photon absorption, our refined heat capacity derivations predict that ultrasmall nanosilicates in the ISM could be heated by 35–80 K more than previously expected.

Until now it has been impossible to directly confirm the presence of ultrasmall nanosilicates in the ISM. However, the James Webb Space Telescope (JWST) now has highly sensitive instruments covering an ideal range of wavelengths to observe nanosilicate IR emission in the ISM.^19^ Our results should allow for more accurate predictions of the IR emission characteristics of stochastically heated nanosilicate grains and could thus be important for accurately interpreting upcoming JWST observations of the ISM.

## Computational Methodology

Optimized nanosilicate structures and their harmonic vibrational spectra were obtained using DFT calculations with an all-electron light-tier 1 numerical atom-centered orbital basis set employing the Fritz Haber Institute ab initio molecular simulations package (FHI-AIMS).^20^ In previous studies it has been shown that this basis set has a quality comparable to valence triple-ζ plus a polarization Gaussian-type orbital basis set.^21,22^ The PBE0^23^ exchange-correlation functional was used in all the calculations. Previous works have shown that this functional is able to accurately reproduce the IR spectra of bulk crystalline olivine^24^ (forsterite) and pyroxene^25^ (enstatite), and the IR spectra of both pyroxene and olivine monomers.^26^

Once the vibrational modes have been calculated for each nanosilicate, we assume that the grain is in thermal equilibrium at a given temperature, *T*, and use standard statistical mechanics^27^ to obtain the average energy, *E̅*(*T*), from
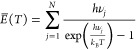
1where *N* is the number of vibrational frequencies of the grain, *ν*_*j*_ are the vibrational frequencies, and *k*_B_ is the Boltzmann constant. By differentiating the expression for average energy in (eq 1) with respect to *T*, one can then obtain the heat capacity of the grain:
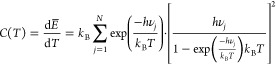
2

In this work we study three sizes of ultrasmall silicate grains: (a) dimers of pyroxene (i.e., (MgSiO_3_)_2_ with 10 atoms) and olivine (i.e., (Mg_2_SiO_4_)_2_ with 14 atoms), (b) 35-atom nanograins of pyroxene (i.e., (MgSiO_3_)_7_) and olivine (i.e., (Mg_2_SiO_4_)_5_), and (c) 70-atom nanograins of pyroxene (i.e., (MgSiO_3_)_14_) and olivine (i.e., (Mg_2_SiO_4_)_10_). The structures of all nanosilicate clusters were found by dedicated global optimization searches following the approach described in our previous work.^18^ Specifically, for the 35-atom and 70-atom nanosilicates, we used the lowest energy structure from these searches. For the pyroxene and olivine dimer species, we employed both the previously reported global minima and also the next two higher energy isomers in each case. We note that the low energy 70-atom pyroxene structure and the metastable dimer species were not reported previously, and their structures are provided in the Supporting Information.

## Results and Discussion

### Vibrational Mode Spectra

**Figure 1:**
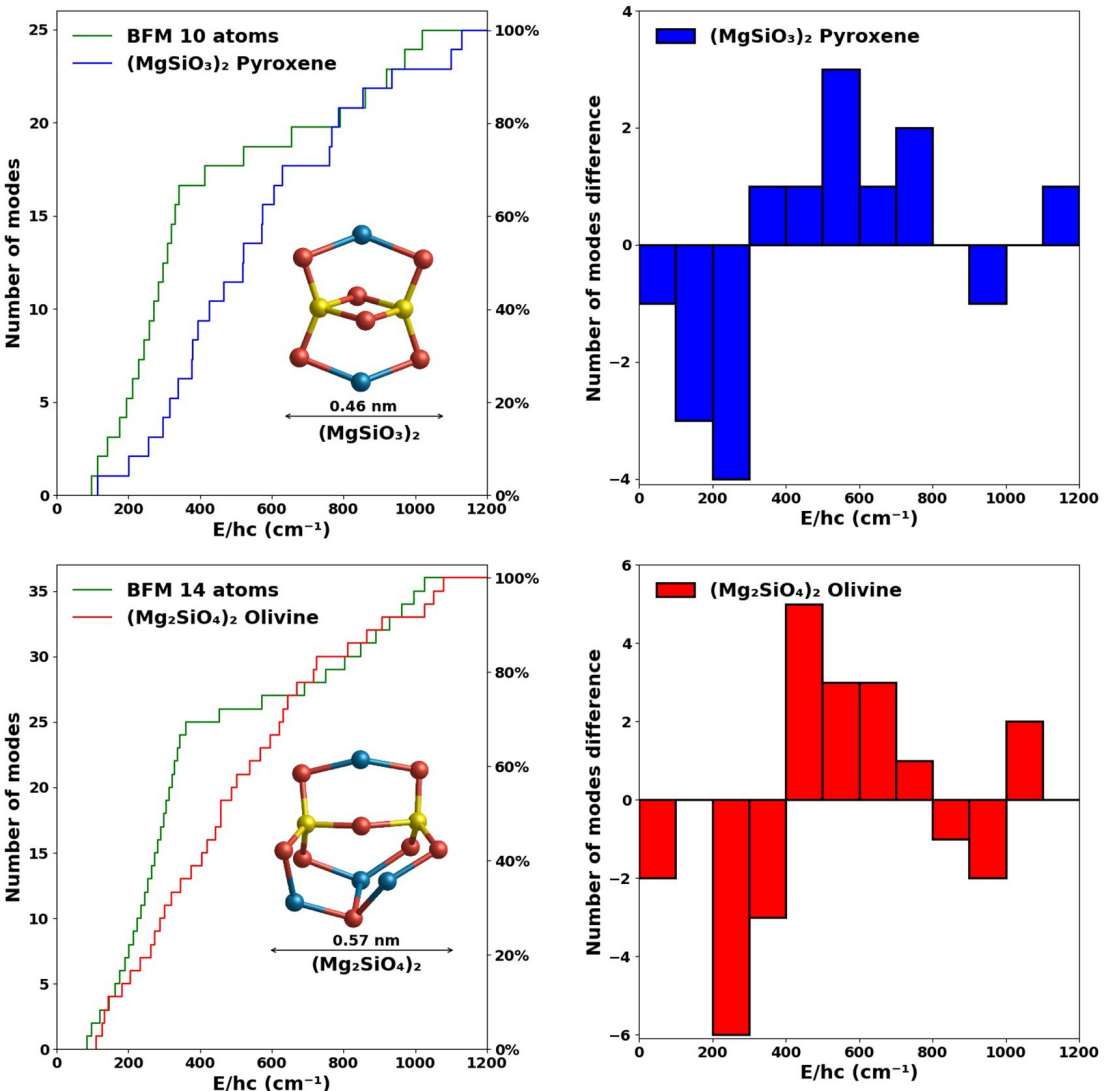
Left: Comparison of the cumulative vibrational mode spectra of pyroxene (blue, upper) and olivine (red, lower) dimers with respect to the corresponding BFM silicate vibrational spectra (green). Right: Differences in number of modes between the DFT and BFM spectra with respect to binned frequency in ranges of 100 cm^–1^. Positive (negative) differences indicate that the DFT-derived spectra have more (fewer) vibrational modes in that bin. Atom color code: red, oxygen; blue, magnesium; yellow, silicon.

**Figure 2:**
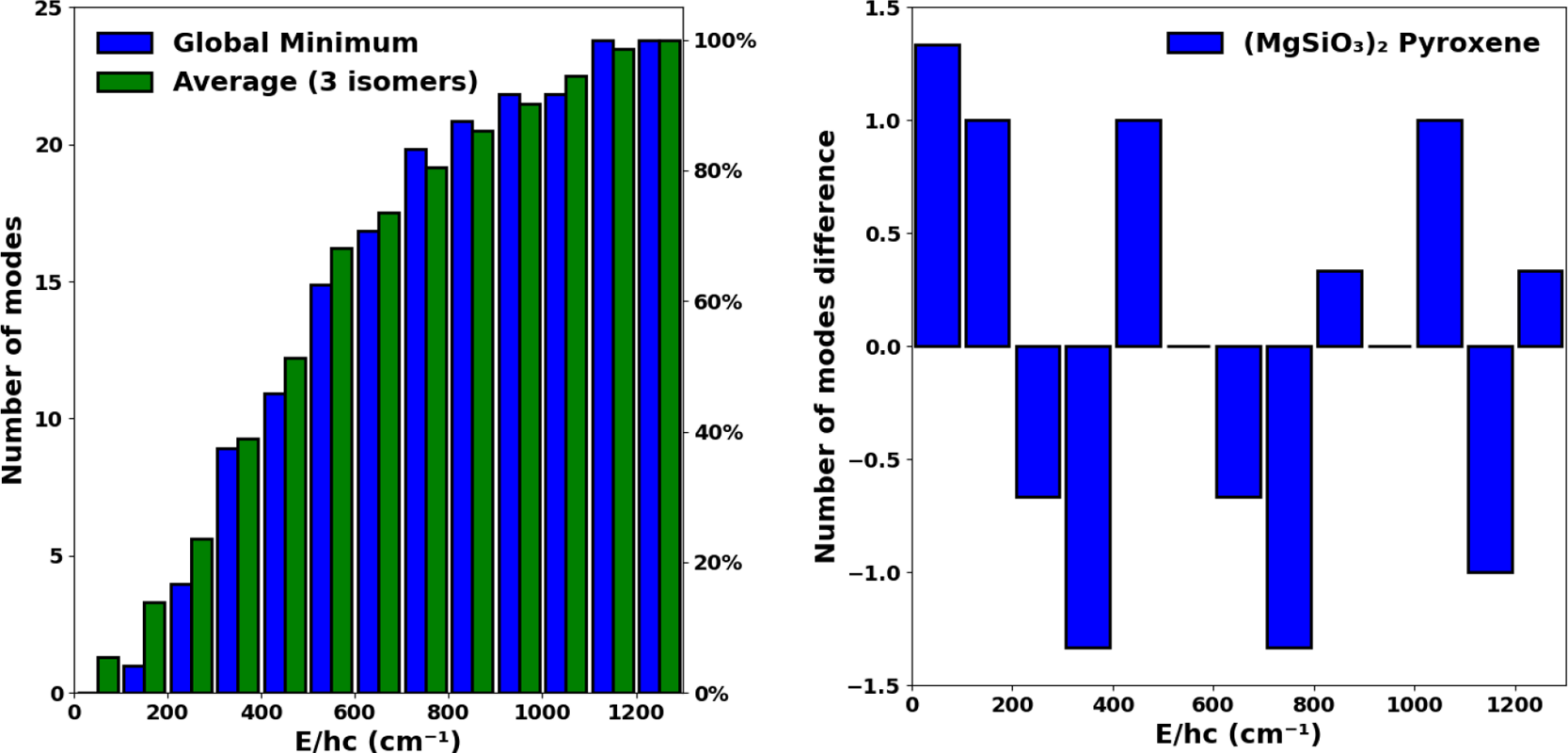
Left: Comparison between the cumulative vibrational spectra of the global minimum pyroxene dimer (blue) and the averaged spectra of three pyroxene dimer isomers (green). Right: The respective difference in the number of modes in each bin (i.e., global minimum vs average). A positive (negative) number of modes difference indicates that the averaged spectrum has more (fewer) vibrational modes in that bin.

In Figure 1 we compare our DFT-calculated vibrational mode spectrum of the lowest energy pyroxene and olivine dimers with that derived from the BFM. In Figure 2 we can clearly see that the BFM spectra overestimates the number of vibrational modes that are found at lower frequencies (approximately 200–600 cm^–1^) with respect to our more realistic model, in which the vibrations are more evenly distributed throughout the whole frequency range. This effect is observed in both pyroxene and olivine dimers, but is more noticeable in the case of pyroxene. In both cases, around 66% of the vibrational modes are found between 0 and 400 cm^–1^ in the BFM spectra. However, in our directly calculated spectra, we do not reach 66% of the modes until a frequency of ∼700 cm^–1^. This difference can be more explicitly seen by summing the number of frequencies found in regular bins of 100 cm^–1^ and comparing the number of frequencies in each bin for each spectrum. From the resulting histograms (see Figure 1, right), we can see that the number of vibrations in the BFM spectra are relatively overestimated in the region between 0 and 300–400 cm^–1^ and underestimated in the region between 400 and 800 cm^–1^. For higher frequencies, all spectra converge and the differences are small.

**Figure 3:**
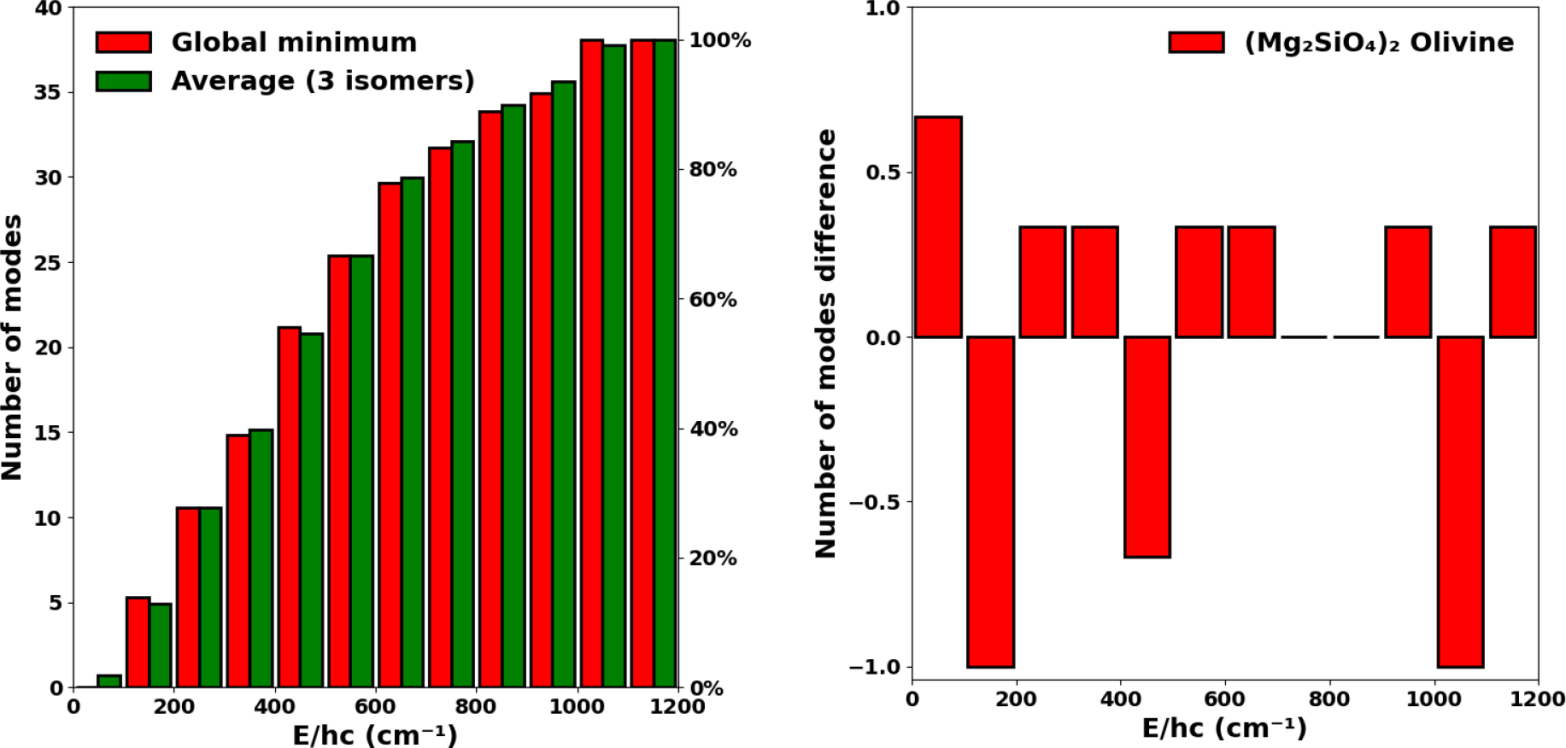
Left: Comparison between the cumulative vibrational spectra of the global minimum olivine dimer (red) and the averaged spectra of three olivine dimer isomers (green). Right: The respective difference in the number of modes in each bin (i.e., global minimum vs average). A positive (negative) number of modes difference indicates that the averaged spectrum has relatively more (fewer) vibrational modes in that bin.

We note that in our previous work we established that increasing the temperature of silicate dimers led to small shifts in the vibrational frequencies as compared to the 0 K harmonic frequencies.^26^ Relatively elevated temperatures (∼800 K) can induce small conformational structural changes in such ultrasmall silicate species.^26,13^ Higher temperatures will lead to a more significant structural isomerization and eventually total melting. To estimate the effect of isomerization, we compare the vibrational spectra of the global minima silicate dimers (i.e., those from Figure 1) with vibrational spectra derived from averaging the number of vibrational modes from a set of three low energy isomers for both pyroxene and olivine dimers. To obtain the average of the spectra, we divide each spectrum into bins of 100 cm^–1^ and average the total number of vibrational modes per isomer in each bin (see Figures 2 and 3).

The comparisons in Figures 2 and 3 show that the resulting vibrational spectra do not change significantly when considering either the lowest energy nanosilicate structure or multiple low energy isomers for either silicate stoichiometries. Specifically, in any 100 cm^–1^ bin, the maximum difference in the number of modes between the two spectra is always found to be less than two. Considering this result, in the rest of the study we shall only consider the spectra obtained from the lowest energy nanosilicate structure for each size and stoichiometry considered.

So far, we have considered only extremely small molecular silicate species. With increasing size, it may be expected that our bottom-up calculated vibrational spectra would better match the BFM vibrational spectra. To begin to answer this question, we obtain the vibrational spectra of larger nanosilicates containing 35 and 70 atoms with both pyroxene and olivine compositions.

**Figure 4:**
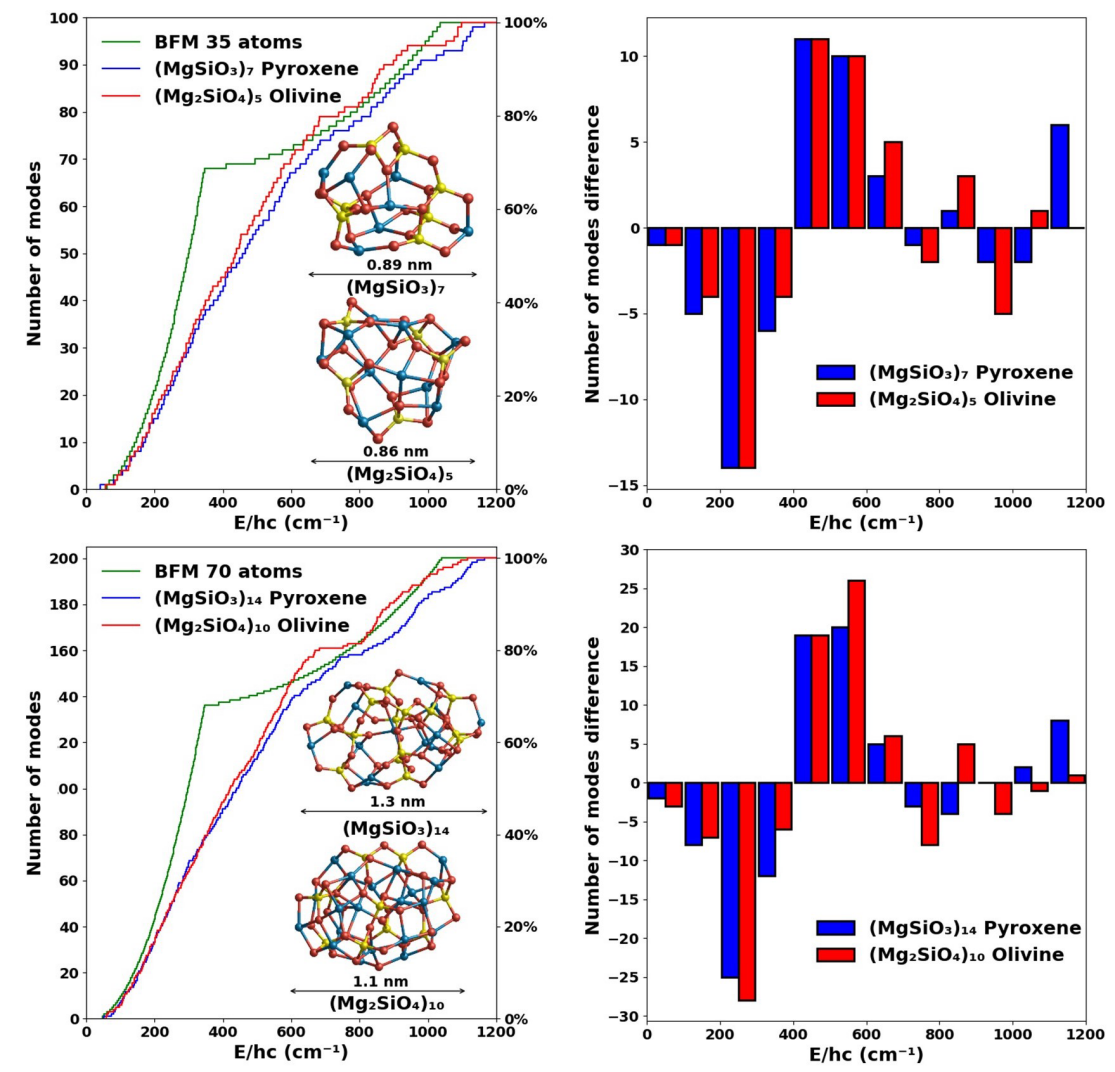
Left: Comparison of the cumulative vibrational mode spectra of pyroxene (blue) and olivine (red) nanosilicates containing 35 (upper) and 70 atoms (lower) with respect to the corresponding BFM vibrational spectra (green). Right: Differences in number of modes between the DFT and BFM spectra with respect to binned frequency ranges of 100 cm^–1^. Positive (negative) differences indicate that the DFT-derived spectra have more (fewer) vibrational modes in that bin. Atom color code as in Figure 1.

From Figure 4 (left), we see that the vibrational spectra for 35-atom and 70-atom nanosilicates follow a very similar tendency as found for the case of the silicate dimers. For both sizes considered, the increase in the number of modes with respect to frequency for both pyroxene and olivine follows a very similar tendency up to ∼700 cm^–1^, where they show the greatest difference with the fitted spectra. In the BFM spectra 66% of the vibrational modes are found between 0 and 400 cm^–1^, whereas in our bottom-up calculated spectra we need to reach 700 cm^–1^ before the same proportion of modes are found. In Figure 4 (right) we show the binned difference in the number of vibrational modes between the bulk-fitted spectra and our calculated spectra. As for the dimers, we can again observe that the BFM spectra begin with an overestimation of the number of vibrational modes, followed by an intermediate region in which the vibrational modes are underestimated with respect to our calculated spectra. For the higher frequency region (>700 cm^–1^), our calculated spectra, for all nanosilicate sizes, and the BFM spectra match quite well. Olivine species show a tendency to have slightly more modes at lower frequencies (approximately <700 cm^–1^) than the pyroxene species of the same size and vice versa at higher frequencies. However, both pyroxene and olivine nanosilicates have a significantly lower number of modes in this frequency range than the BFM spectra.

Generally, our calculated vibrational mode spectra for all pyroxene and olivine nanosilicates show a relatively smooth monotonic increase in the number of modes with increasing frequency. In particular, none of our nanosilicates display a clear two-phase pattern to their cumulative vibrational spectra, with an abrupt change at ∼300 cm^–1^, as used in the BFM spectra. It is of note, however, that for the largest size of 70 atoms (*r* ≈ 0.5/0.6 nm), we observe some indications of an emerging change in slope of the cumulative vibrational spectra near to 700 cm^–1^ (12.5 μm). We speculate that this may be linked to the emergence of the separate 10 and 18 μm IR vibrational bands observed in larger silicate dust particles.^7^

Finally, we note that all our vibrational spectra are derived using the harmonic approximation. Any vibrational modes exhibiting anharmonicity tend to shift to slightly lower frequencies (with respect to purely harmonic modes) with increasing temperature. We have previously found that this effect is small in nanosilicates and only becomes apparent above at approximately 400 K.^26^ Assuming a relatively large anharmonic downshift of all our harmonic frequencies by 5%, we find that our final results regarding the stochastic heating are essentially unchanged (see SI for details).

### Heat Capacity

**Figure 5:**
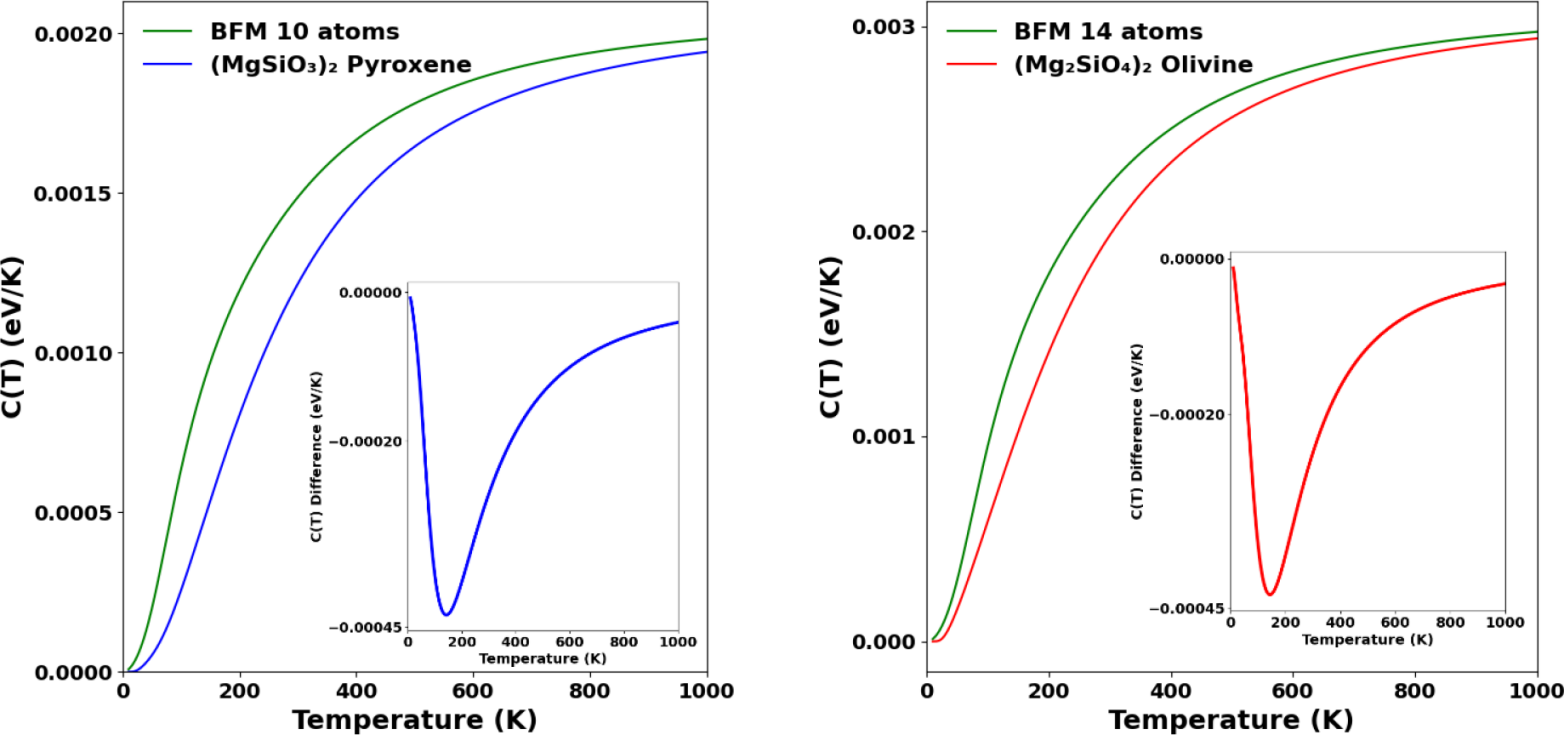
Specific heat capacity for pyroxene (left) and olivine (right) dimers compared with BFM (green). Inset plots show the heat capacity difference between the two respective *C*(*T*) curves.

**Figure 6:**
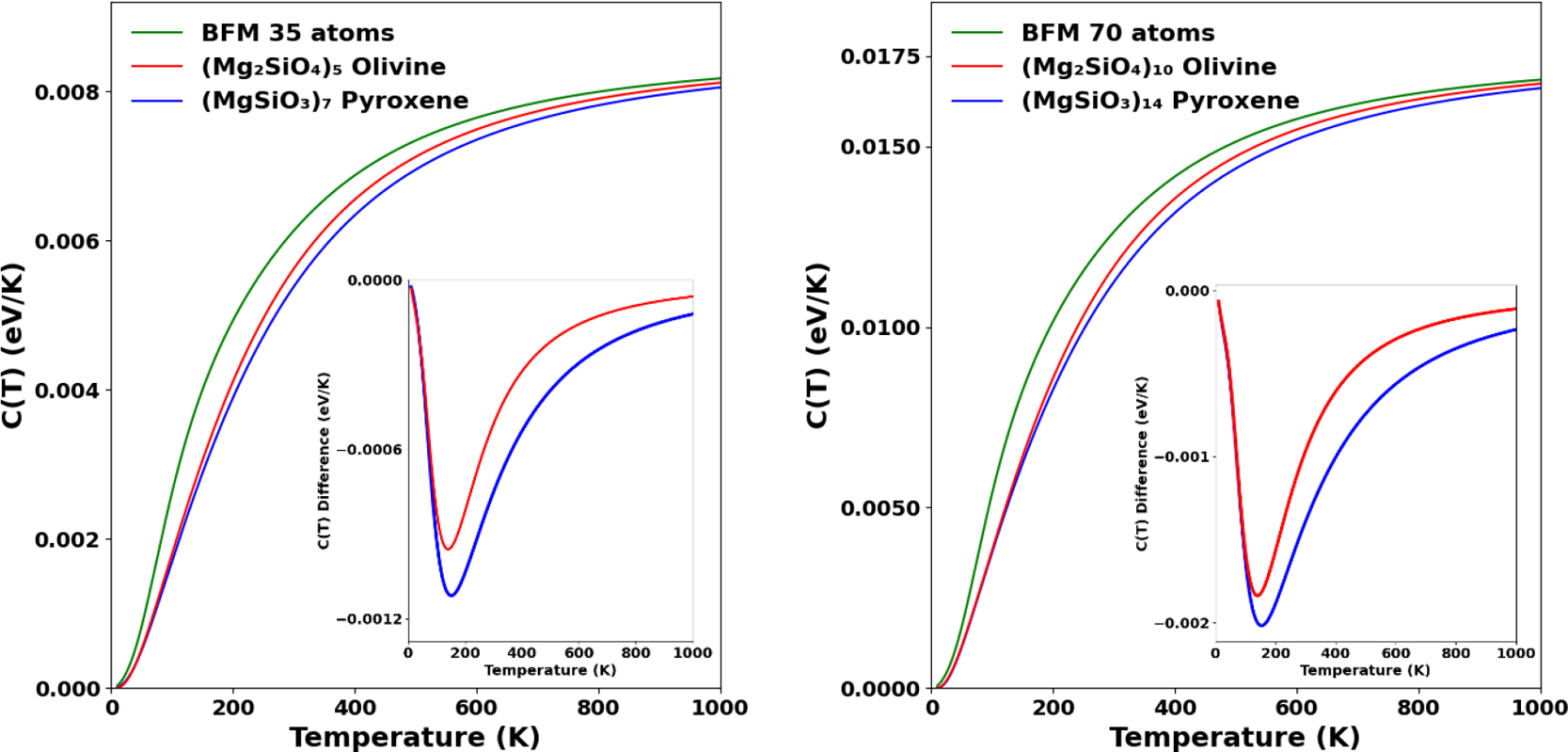
Specific heat capacity for pyroxene (blue) and olivine (red) nanosilicates with 35 (left) and 70 atoms (right) compared with BFM (green). Inset plots show the difference between the two respective *C*(*T*) curves.

The heat capacity, *C*(*T*), of a grain can be derived from the spectrum of its vibrational modes as described above (see eq 2). As we have seen, there are significant differences between our DFT-calculated vibrational mode spectra and those from the BFM, which will thus influence the corresponding *C*(*T*) values. As this vibrational difference is especially notable in the low frequency region, we expect that the corresponding largest *C*(*T*) differences would be at lower temperatures. In Figure 5 we show the *C*(*T*) curves derived from the vibrational spectra shown in Figure 1 for the silicate dimer species. We see that the heat capacity derived from our vibrational modes for the pyroxene and olivinic dimers is generally lower than the BFM heat capacity. This difference has its maximum around 150 K and is larger for the pyroxene stoichiometry. In Figure 6 we show the *C*(*T*) curves for 35-atom and 70-atom nanosilicates, where we also observe the corresponding differences with the BFM as found in the case of the dimers.

The differences between the calculated specific heat capacities in Figures 5 and 6 can be simply explained from the corresponding differences in the vibrational mode spectra in each case. Above, we noted that the BFM vibrational spectra always overestimates the number of modes in the lower frequency region with respect to our DFT-calculated vibrational spectra. For nanosilicates having the same number of atoms, those with more modes at lower frequencies are able to distribute modest energy increases throughout more degrees of freedom, thus, resulting in a relatively lower temperature increase (i.e., a larger heat capacity).

**Figure 7:**
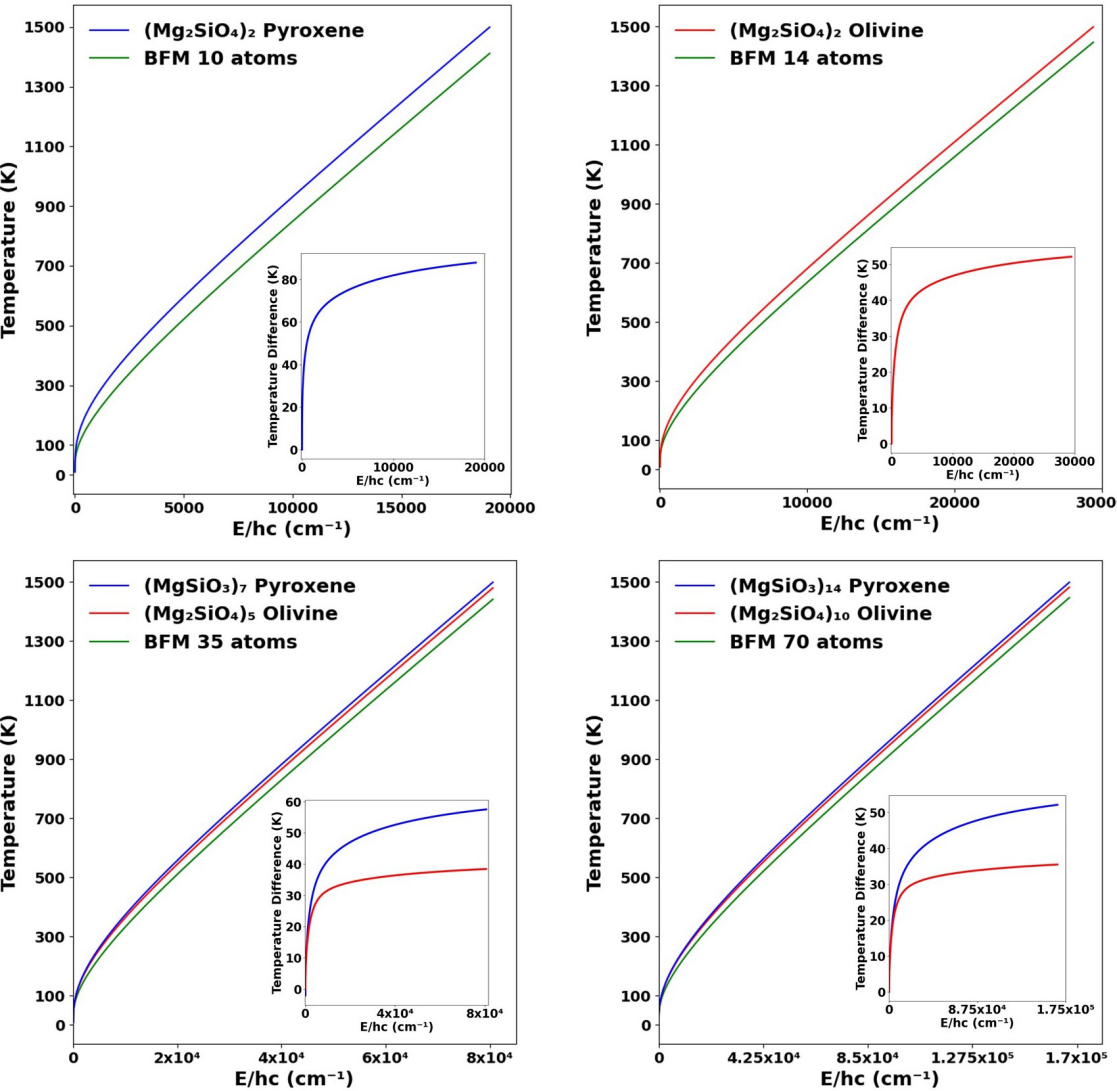
Temperature vs average energy plots for nanosilicates derived from DFT-calculated vibrational spectra (pyroxenes, blue; olivines, red) and from the BFM vibrational spectra (green) for different sizes. Insets show the difference between the DFT-derived values and the BFM-derived values (pyroxenes, blue; olivines, red).

The heat capacity plays an important role in calculating how these ultrasmall silicates absorb and emit radiation in the ISM and thus how they are affected by stochastic heating.^5,16,17,6^ Our results show that the BFM vibrational spectra lead to overestimates of *C*(*T*) with respect to heat capacities derived from accurate directly computed vibrational spectra for ultrasmall silicates. For such species, the immediate temperature rise induced by absorbing a UV photon in the ISM can be calculated using eq 1. In Figure 7 we show temperature versus average energy plots for nanosilicates for a range of nanosilicate stoichiometries and sizes. The energy axes in each case in Figure 7 cover the energies of typical UV photons in the ISM. We truncate the plots at a temperature at 1500 K, which is likely to be near the melting temperature of the largest nanosized silicate grains considered. We note that even the smallest nanosilicate species can withstand 800 K without fully melting.^13,26^

The plots in Figure 7 show that the smallest silicate dimers would be readily heated to close to 1000 K by relatively low energy visible photons (∼1.5 × 10^4^ cm^–1^/∼1.2 eV). In such a scenario, our results show that BFM-based calculations underestimate the photon-induced instantaneous temperature increase by 50–80 K (see insets in the upper two plots in Figure 7). For larger nanosilicate grains with 35–70 atoms, higher energy UV photons are required to achieve a similar ∼1000 K heating. In such cases, the BFM underestimation is ∼35 K for olivine and ∼55 K for pyroxene nanosilicates (see insets in the lower two plots in Figure 7). For small grains the interval between successive visible/UV photon absorption is relatively long compared with the time for IR emission,^5,16^ and stochastically heated ultrasmall nanosilicates will spend most of their time close to that of their ISM environment. However, considering that the total spectral flux is proportional to *T*^4^, these temperature corrections could lead to noticeable blue shifting in the emission spectra of the, presumably, very high population of ultrasmall nanosilicates in the ISM.

**Figure 8:**
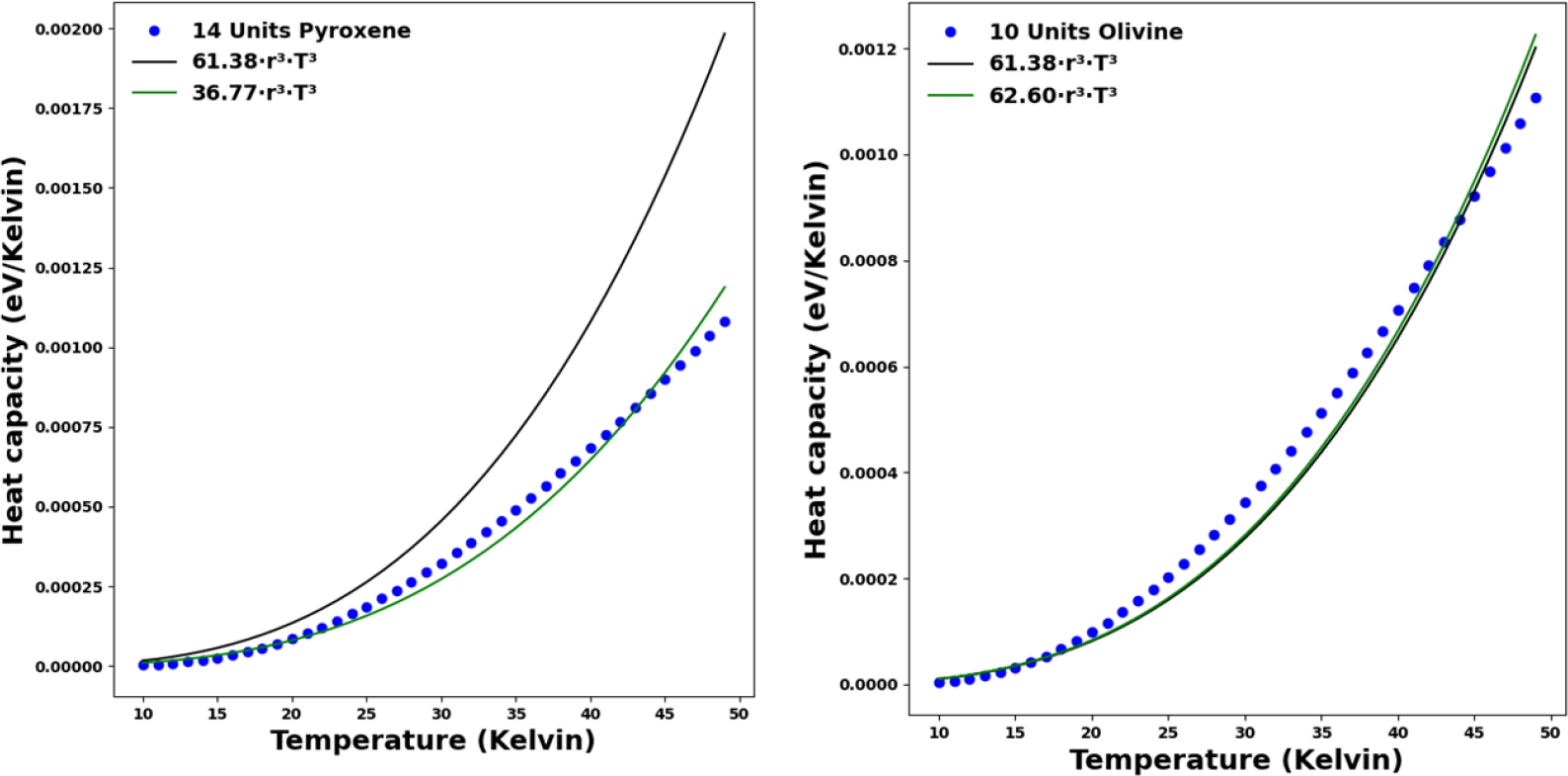
Comparison of the low-temperature BFM-like fitted *C*(*T*) used in refs^16 and 17^ (black solid line) and our DFT-derived *C*(*T*) values for 70-atom pyroxene (left) and olivine (right) nanosilicates (blue data points). We include an approximate fit to our data (green solid line) for comparison with the BFM-like fit.

Finally, we note that for larger nanograins with radii of a few nanometers, stochastic heating is predicted to produce relatively milder heating of a few 10s of Kelvin.^5,16,17^ Such predictions are typically made on the basis of low temperature (0–50 K) approximations to the BFM-derived *C*(*T*).^16,17^ In Figure 8 we compare our DFT-derived *C*(*T*) data with the low temperature BFM-like fitted *C*(*T*) used in refs (16 and 17) for 70-atom nanosilicates. Fitting our data with an Ar^3^T^3^ expression, we find that for olivines there is a good match between our data and the low temperature fit. However, for pyroxenes, the fit to our data is significantly different to the low temperature BFM-like fit. Although the studies in refs (16 and 17) relate to larger nanosilicates than we consider, these results clearly show that there is a limit to the size-dependent applicability of the low-temperature limiting fit of *C*(*T*) for nanopyroxenes. Perhaps surprisingly, however, this fitted expression appears to hold for very low temperature stochastic heating of even ultrasmall olivine nanosilicates.

## Conclusions

We calculate accurate vibrational spectra for ultrasmall nanosilicates (10–70 atoms) with stable low energy structures and with both Mg-rich pyroxene and olivine stoichiometries using quantum chemical DFT calculations. Such spectra are known to be significantly affected by the extreme small size of these species and quite distinct from the vibrational spectra of bulk silicates. Comparing our data with vibrational mode spectra that were empirically fitted to reproduce bulk-like *C*(*T*) curves for bulk silicates (BFM), for example, we find significant differences, especially at low frequencies (≤700 cm^–1^). Using our ab initio vibrational spectra, we also compute heat capacities of a range of nanosilicates from standard statistical mechanics. Generally, our *C*(*T*) values are found to be lower than those derived using the BFM, especially around 150 K. These differences imply that the instantaneous photon-induced temperatures of relatively hot stochastically heated small nanograins in the ISM would be 35–80 K higher than previously expected. Our accurately derived *C*(*T*) values could also have implications for relatively low-temperature stochastic heating of larger nanosilicate grains. Generally, our study shows the power of an accurate bottom-up quantum chemical approach for directly calculating the properties of nanoscale dust grains. Our results may provide useful insights for improving the modeling of the IR emission of the likely high population of ultrasmall nanosilicates in the ISM, which hopefully will be confirmed by upcoming JWST observations.

## Supporting Information Available

Estimated maximal effect of anharmonicity on calculated *C*(*T*); Cartesian coordinates of metastable silicate dimers (PDF)
