# Supplementary material for: Efficiency of Interstellar Nanodust Heating: Accurate Bottom-up Calculations of Nanosilicate Specific Heat Capacities
Source: J Phys Chem A. 2022 Jun 8;126(24):3854–62. doi: 10.1021/acs.jpca.2c02199 (PMC9881164; doi:10.1021/acs.jpca.2c02199)
Supplement: Supplementary file 1 — jp2c02199_si_001.pdf [file jp2c02199_si_001.pdf]

Supporting Information for:

Efficiency of Interstellar Nanodust Heating: Accurate Bottom-up Calculations of Nanosilicate Specific Heat Capacities

Joan Mariñoso Guiu,[1] Stefan T. Bromley[1,2,*]

[1]*Departament de Ciència de Materials i Química Física & Institut de Química Teòrica i Computatcional (IQTCUB), Universitat de Barcelona, c/ Martí i Franquès 1-11, 08028 Barcelona, Spain*

[2]*Institució Catalana de Recerca i Estudis Avançats (ICREA), Passeig Lluis Companys 23, 08010 Barcelona, Spain*

* Corresponding author: s.bromley@ub.edu

S1 Effect of anharmonicity

To estimate the maximal effect of anharmonic frequency shifts, we took the largest temperature-induced downshift observed for some frequencies in small nanosilicates in our previous work (~5% at 800K)[1] and applied it to all frequencies for the 35-atom pyroxene (i.e. $(MgSiO_3)_7$) and olivine (i.e. $(Mg_2SiO_4)_5$) species. In Fig. S1a we plot the resulting "maximally anharmonic" vibrational mode spectra and the corresponding BFM mode spectrum for 35 atoms. Overall, the shifted spectral distributions do not change significantly with respect to the harmonic case. With respect to the bare harmonic spectra, the anharmonic pyroxene spectrum now coincides with the BFM spectrum between 700 cm^{-1} and 1000 cm^{-1}, but the olivine spectrum shows a greater overall mismatch with the BFM spectrum (see also Fig. 4 in the main text).

The resultant effect of this frequency downshift on the specific heat capacity with respect to that obtained from is very small. Specifically, in Fig. S2 we show the difference in the specific heat capacity resulting from downshifted mode spectra for the 35-atom pyroxene and olivine species with respect to those derived from the corresponding harmonic mode spectra. The resultant difference in the temperature of these nanosilicates (with respect to energy) compared to that calculated using the harmonic vibrational spectra is shown in Fig. S3. Here, the temperatures are maximally affected by 3.5-4.0% for very low energies, but with increasing energy the difference in temperature between the maximally anharmonic and harmonic approaches decreases significantly.

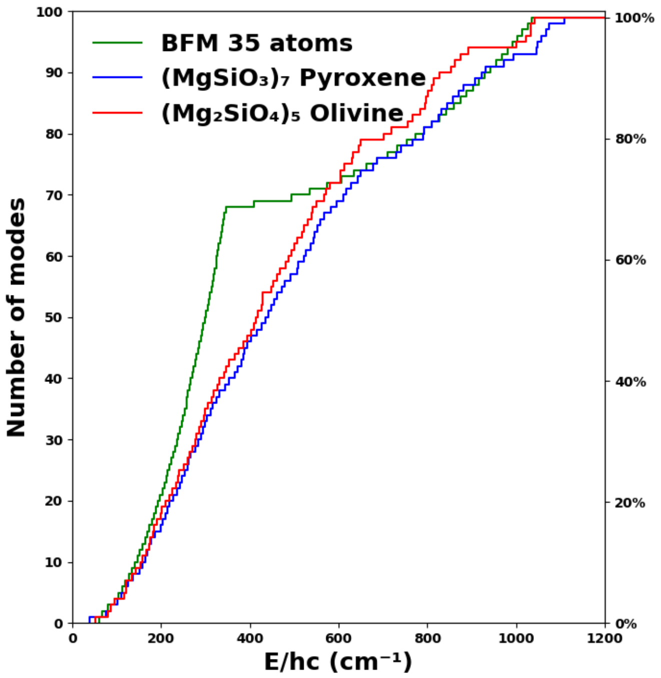

Figure S1 Vibrational mode spectra for 35-atom pyroxene (i.e. $(MgSiO_3)_7$) and olivine (i.e. $(Mg_2SiO_4)_5$) nanosilicates after application of a 5% downshift in all frequencies (i.e. maximally anhamonic) with respect to the corresponding BFM spectrum.

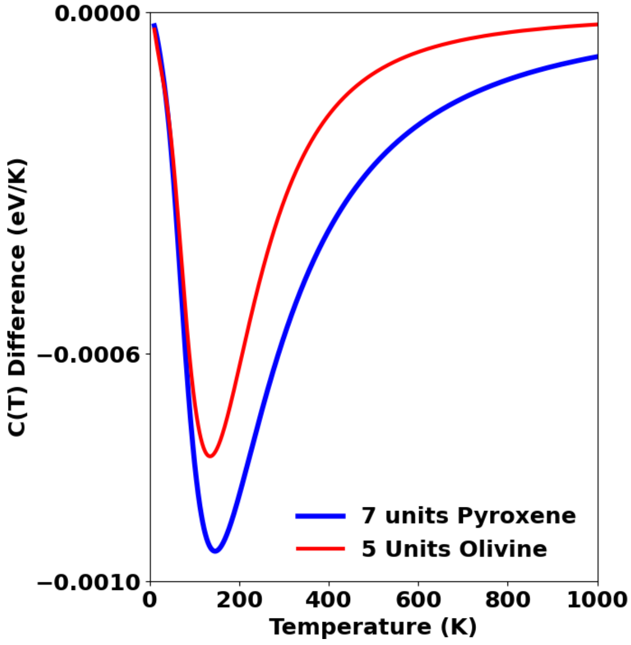

Figure S2. Difference in $C(T)$ calculated using the maximally anharmonic vibrational spectra with respect to C(T) based on the harmonic vibrational spectra for 35-atom pyroxene (i.e. $(MgSiO_3)_7$) and olivine (i.e. $(Mg_2SiO_4)_5$) nanosilicates.

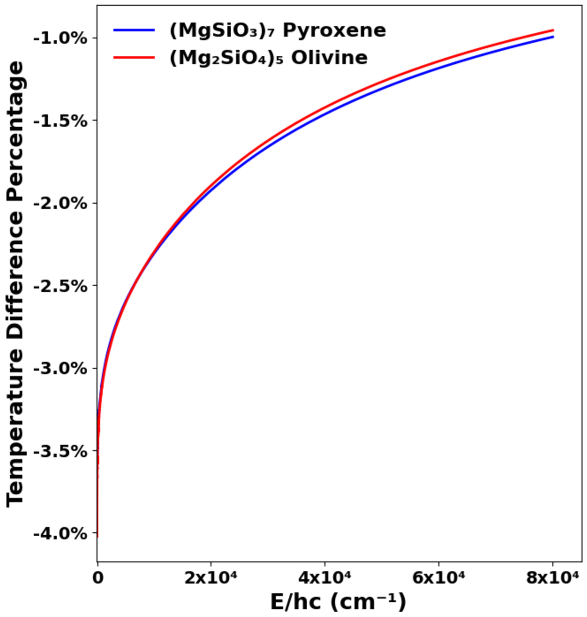

Figure S3. Percentage change in temperature of 35-atom pyroxene (i.e. $(MgSiO_3)_7$) and olivine (i.e. $(Mg_2SiO_4)_5$) nanosilicates with respect to energy as calculated using the maximally anharmonic vibrational spectra with respect to the temperature calculated using the harmonic vibrational spectra.

S2 Cartesian coordinates of metastable silicate dimers

Pyroxene isomers

Isomer 2:

10

$(MgSiO_3)_2$ 2nd isomer

Mg	-2.80820614	2.68486963	0.91914789
Mg	-0.67664681	-0.24206989	-1.10450779
Si	-2.6424027	1.32478257	-1.18982681
Si	-1.93422345	0.32795385	0.99748922
O	-0.87627754	-0.84707909	0.74418243
O	-3.42967538	2.69561035	-0.93486856
O	-2.41357017	1.26502003	2.2042514
O	-1.8921193	0.5836224	-2.39475733
O	-3.20918911	0.16043695	-0.10163372
O	-1.26969941	1.56337721	-0.08992473

Isomer 3:

10

$(MgSiO_3)_2$ 3rd isomer

Mg	0.65423788	-0.37484312	-0.81969484
Mg	-1.83203011	1.74348272	0.06482119
Si	-2.3451357	-0.78611903	-1.79554438

Si	0.39366047	1.23918846	1.05527673
O	0.12726478	1.57761033	-0.50453472
O	1.33384967	-0.0155445	1.08081398
O	-0.79487457	-1.18390255	-1.60252602
O	-3.30700587	-1.52584862	-2.72505024
O	-0.82579967	1.82514109	1.84852549
O	-2.75968588	0.49022221	-0.90243018

Olivine isomers:

Isomer 2:

14
$(Mg_2SiO_4)_2$ 2nd isomer

O	0.96548026	-2.63651933	1.59906559
Si	-0.3235138	-2.10788641	0.79229676
O	0.41979945	-0.76024505	-0.00021708
O	-1.14878965	-2.89032507	-0.34891871
O	-0.07724183	1.72735189	0.55083267
Mg	-1.42201277	0.53925945	1.17513145
Si	1.10216667	0.81686245	-0.20787842
O	-1.55780831	-1.31002345	1.58920909
O	2.51789689	0.54885824	0.51021163
Mg	-1.00545578	1.45834564	-1.35079005
O	-2.40804361	0.42673481	-0.56022127
O	0.84846113	1.20808136	-1.75002985
Mg	2.13675839	-1.24457064	1.03049939
Mg	-2.43383004	-1.47360089	-0.3493842

Isomer 3:

14
$(Mg_2SiO_4)_2$ 3rd isomer

O	-0.93595215	-0.52041685	2.11523282
Si	0.38778406	-0.95563131	1.32993186
O	3.30436814	-0.07787728	-0.04750157
O	-1.36066389	2.12882746	0.09014434
O	0.45673887	0.11736719	-0.05180572
Mg	2.69664036	-1.68133417	0.86241713
Si	0.19593226	1.86882173	-0.14424553
O	0.84969027	2.20034048	1.36125614
O	0.88944655	-2.34939793	0.68005857
Mg	-1.10883503	1.34806242	1.84124148
O	1.77186775	-0.55672583	2.21876054
O	1.15772429	2.12873678	-1.40287505
Mg	1.98915125	0.40544978	-1.33508867
Mg	2.39620028	1.03648552	1.37511267

[1] Mariñoso Guiu, J.; Macia Escatllar, A.; Bromley, S. T. How Does Temperature Affect the Infrared Vibrational Spectra of Nanosized Silicate Dust? *ACS Earth Space Chem*. **2021**, 5, 4, 812–823. DOI: 10.1021/acsearthspacechem.0c00341
